# Empyema and Respiratory Failure Secondary to Nephropleural Fistula Caused by Chronic Urinary Tract Infection: A Case Report

**DOI:** 10.1155/2012/595402

**Published:** 2012-11-13

**Authors:** G. H. Jones, H. R. Kalaher, N. Misra, J. Curtis, R. J. Parker

**Affiliations:** ^1^School of Medicine, Mersey Deanery, Regatta Place, Brunswick Business Park, Summers Road, Liverpool L3 4BL, UK; ^2^Department of Respiratory Medicine, Aintree University Hospitals, NHS Foundation Trust, Lower Lane, Liverpool L9 7AL, UK; ^3^Department of Surgery, Aintree University Hospitals, NHS Foundation Trust, Lower Lane, Liverpool L9 7AL, UK; ^4^Department of Radiology, Aintree University Hospitals, NHS Foundation Trust, Lower Lane, Liverpool L9 7AL, UK

## Abstract

We report a case of nephropleural fistula causing empyema and respiratory failure in a 68-year-old gentleman with a long history of urological problems including recurrent nephrolithiasis and urinary tract infections. He was admitted with sepsis, a productive cough, pyuria, and reduced breath sounds over the left hemithorax. Radiological imaging revealed a fistulous connection between a left-sided perinephric abscess and the pleural space. He was commenced on broad spectrum intravenous antibiotics but developed progressive respiratory failure requiring intensive care admission. Urinary and pleural aspirates cultured facultative anaerobic pathogens with identical resistance patterns. Drainage of thoracic and perinephric collections was carried out, allowing him to be extubated after 24 hours and discharged home after 18 days on an extended course of oral antibiotics. Left nephrectomy is now planned after a period of convalescence. Empyema developing in patients with known urolithiasis should alert the treating physician to the possibility that a pathological communication has formed especially if typical urinary tract pathogens are cultured from respiratory sampling.

## 1. Introduction

Pulmonary complications, especially pleural effusions, are not uncommonly associated with subdiaphragmatic pathology; however, empyema that forms secondary to a fistula extending between the urinary tract and thorax is a very rare clinical entity.

In modern healthcare settings the majority of nephropleural (NPF) and nephrobronchial fistulae (NBF) form in patients with known nephrolithiasis, as it is now rare for chronic suppurative infections, such as tuberculosis, to go untreated for the prolonged periods required to allow a fistula to become established.

Pulmonary symptoms can dominate the clinical picture in cases of NPF when the underlying association with retroperitoneal sepsis is overlooked, and the delay in the correct diagnosis being established can allow recurrent episodes of suppurative thoracic infection to occur, significantly decreasing the chances of preserving the affected kidney's viability [1]. 

The general management of NPF and NBF includes antimicrobial therapy which may be required for an extended course, drainage of purulent material from both thorax and abdomen prior to definitive treatment by nephrectomy of the infected kidney.

Most patients with NPF present with signs of sepsis from their empyema and perinephric collections; although pleural effusions may be large, infective symptoms occur early, and therefore it is exceptionally rare for significant respiratory failure to occur.

We report a case of NPF developing in a 68-year-old man with longstanding urological problems who required positive pressure ventilation within 24 hours of admission.

## 2. Case Presentation

A 68-year-old gentleman was admitted to hospital through the emergency department with increasing breathlessness, productive cough, and a four-week history of recurrent left-sided loin pains.

Past medical history was of adenocarcinoma of the sigmoid colon 10 years previously for which he had undergone a curative surgical resection. Intraoperative urethral injury required him to intermittently self-catheterise his bladder and over the subsequent decade he had suffered multiple urological problems including recurrent urinary tract infections, nephrolithiasis, and hydronephrosis, particularly of the left renal collecting system. Five months prior to the index admission a nephrostogram had shown multiple calculi on the left-hand side with a multilayered calcified stone causing compression of the pelvicalyceal system and a separate stone at the level of the vesicoureteric junction, and therefore both an antegrade stent (24 cm JJ stent) and a nephrostomy tube (capped 8 French) were inserted. Despite this intervention he continued to suffer upper urinary tract infections that required treatment with intravenous antibiotics. An isotope renogram (99Tcm MAG-3 with furosemide) performed 2 weeks prior to admission had revealed a drainage curve suggestive of ongoing obstruction and a split renal function of 31% on the left and 69% on the right.

**Figure 1:**
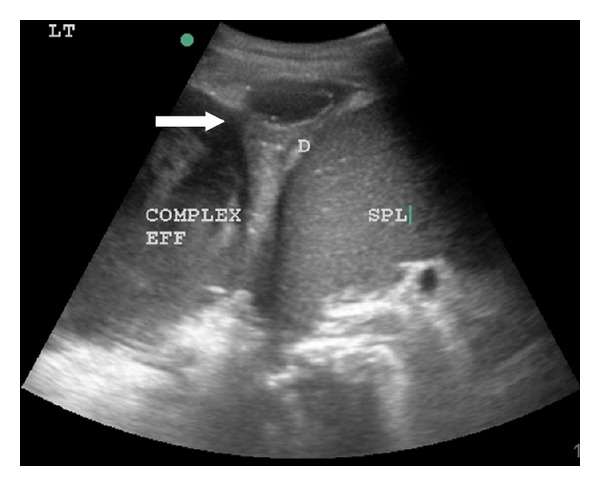
Ultrasound appearance of complex pleural effusion with extension through diaphragm (white arrow). D: diaphragm. SPL: spleen.

On initial assessment he was tachycardic, tachypnoeic, and febrile with reduced breath sounds over the left hemithorax. He was noted to be expectorating malodorous creamy yellow sputum and purulent urine drained on insertion of a Foley catheter. Inflammatory markers were raised (leucocyte count 25.0 × 10^9^/L, C-reactive protein 250 mg/dL), and serum urea and creatinine were within the local normal reference ranges. A moderate-sized left-sided pleural effusion was seen on chest radiograph. Given his extensive urological history a computed tomographic (CT) scan of the renal tract was organised showing a left-sided perinephric collection and a large pleural effusion that had gas-containing areas consistent with an empyema. A respiratory consult was arranged, and bedside ultrasound examination confirmed a complex loculated pleural effusion (see Figure 1).

**Figure 2:**
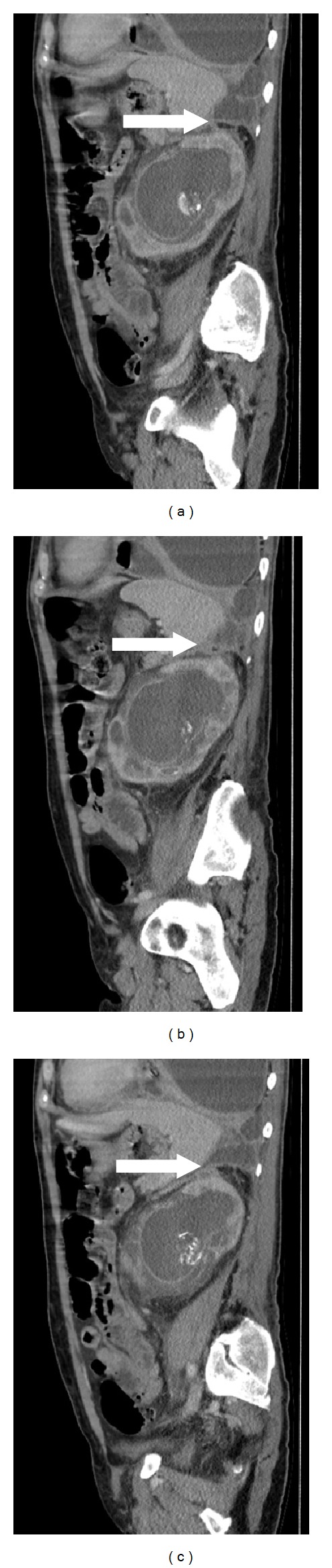
Sagittal CT images showing fistula between left kidney and pleural cavity (white arrow).

**Figure 3:**
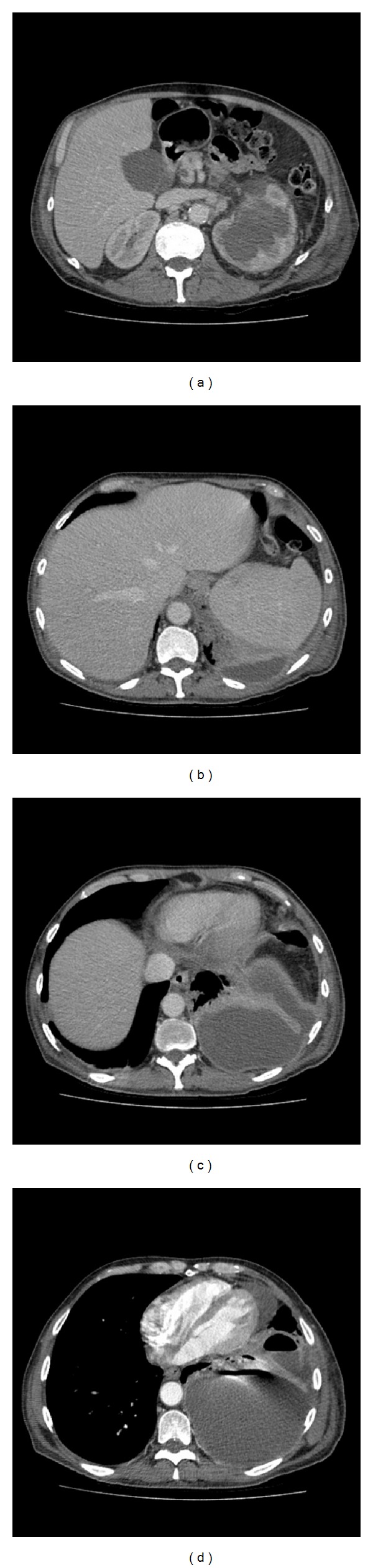
Axial images showing route of nephropleural fistula.

Further discussion with a consultant radiologist about the radiographic appearance of the empyema determined that there was in fact a fistulous tract extending between the retroperitoneal collection and the pleural space (see Figures 2 and 3). Over the following day he exhibited signs of respiratory distress and an urgent chest drain was inserted (24 Fr Thal-Quick, Cook inserted by Seldinger technique under direct ultrasound guidance). Over 1 L of frankly purulent material drained and although his observations initially improved, he continued to require supplementary oxygen therapy and developed progressive respiratory failure. He was therefore transferred to the intensive care unit where he required endotracheal intubation and mechanical ventilation. He was commenced on intravenous piperacillin/tazobactam, and a percutaneous nephrostomy (8 fr locking drain) was performed to drain the left perinephric abscess. Microbiology specimens from both the pleural fluid and urine grew mixed facultative anaerobes (*Escherichia coli* and *Enterococcus faecalis*) with identical resistance patterns. 

Successful extubation was performed after 24 hours of ventilatory support, and he continued to improve, being stepped down from the high dependency unit with good resolution of the empyema radiologically. Prior to discharge to the urological ward and after discussion with the microbiology team, it was decided to follow on from his intravenous antibiotics with an extended course of targeted oral antimicrobial therapy with metronidazole, amoxicillin, and ciprofloxacin. A left nephrectomy is now planned, after a period of convalescence, to deal definitively with the source of the recurrent infection.

## 3. Discussion

Pulmonary manifestations of intra-abdominal pathology are not uncommon, and simple (i.e., noninfected) pleural effusions in particular are associated with a wide range of diseases of subdiaphragmatic origin. Hepatic hydrothorax, nephrotic syndrome, and Meig's syndrome are associated with transudative pleural effusions, while the effusions that develop secondary to conditions such as pancreatitis, subphrenic collections, or as a paraneoplastic phenomenon in cases of abdominal malignancy tend to be exudative in nature. The renal system has a specific pleural effusion associated with it in the form of “urothorax,” a condition where obstructive uropathy leads to extravasated urine crossing the diaphragm via lymphatic channels or congenital perforations [2] and is one of the few causes of an effusion that has both low pH and protein levels. It has been suggested that pulmonary complications develop in up to 20% of renal infections [3] and can include both simple exudative as well as transudative pleural effusions, with case reports suggesting they are more likely to develop during pregnancy [4–6], although this may be due to publication bias with obstetric journals being perhaps more likely to print such cases.

The vast majority of complicated pleural effusions and empyemas develop following pneumonia, and as such the causative organisms tend to be Gram-positive aerobic species (predominantly *Streptococcus sp.* and *Staphylococcus aureus* if acquired in a community or hospital setting, resp. [7]). However in a series of 122 empyemas, 15 cases developed as a result of abdominal pathology of which 5 were directly related to renal tract infection [8]. 

Perinephric infection can be directed towards the thorax if it tracks up the superior attachment of Gerota's fascia where it fuses with the diaphragmatic surface. Congenital malformations of the diaphragm, such as Bochdalek's foramen, may also act to allow retroperitoneal infection to transit into the chest.

Empyema developing secondary to a nephropleural fistula tends to involve those organisms that cause upper urinary tract infections, for example, Gram-negative facultative anaerobes such as *Escherichia coli* and *Proteus mirabilis* [9]—both of which have also been implicated in the development of xanthogranulomatous pyelonephritis, a condition that is reported to be particularly prone to causing fistula that crosses the diaphragm [10–12]. Mycobacterial infections have also been implicated in the formation of pathological communications between the renal tract and thorax [13] and were identified by Aly et al. as aetiological in almost 20% of cases of nephrobronchocutaneous fistula of which there are less than one hundred cases reported in the literature [14]. In areas that have poor access to effective antimicrobial therapies, primary suppurative infections may go untreated for extended periods thereby facilitating the formation of a fistula, whereas in areas with ready access to modern healthcare services, where chronic indolent infections are rare, the literature seems to suggest that the majority of nephropleural fistulas develop in patients with known nephrolithiasis and may also complicate those invasive techniques used in the management of calculus pyonephrosis [15].

Any patient with recurrent or unexplained suppurative thoracic infection with known or suspected renal tract calculus should be investigated to exclude an underlying fistula particularly if typical urinary tract pathogens are cultured on pleural sampling. Initial treatment should include prompt drainage of pyogenic collections from thorax and abdomen with concurrent initiation of appropriate antibiotics—which are likely to be needed for an extended course. Definitive management usually necessitates nephrectomy, often as in the case we described an involved kidney will only have minimal residual function by the time a nephropleural fistula has become established, this is debated and some centres suggest conservative management or stenting as an alternative to surgery [16].

In summary, nephropleural fistula remains such a rare clinical entity that even when physicians have a high clinical index of suspicion and access to modern radiological imaging establishing the diagnosis is a challenge, especially in cases where respiratory symptoms predominate [1] or underlying urolithiasis is clinically silent [17].
